# Genetic Variability in Local and Imported Germplasm Chicken Populations as Revealed by Analyzing Runs of Homozygosity

**DOI:** 10.3390/ani10101887

**Published:** 2020-10-15

**Authors:** Natalia V. Dementieva, Andrei A. Kudinov, Tatiana A. Larkina, Olga V. Mitrofanova, Artyom P. Dysin, Valeriy P. Terletsky, Valentina I. Tyshchenko, Darren K. Griffin, Michael N. Romanov

**Affiliations:** 1 Russian Research Institute of Farm Animal Genetics and Breeding (RRIFAGB)—Branch of the L.K. Ernst Federal Science Centre for Animal Husbandry, Pushkin, St. Petersburg 196601, Russia; dementevan@mail.ru (N.V.D.); kudinov_aa@list.ru (A.A.K.); tanya.larkina2015@yandex.ru (T.A.L.); mo1969@mail.ru (O.V.M.); artemdysin@mail.ru (A.P.D.); valeriter@mail.ru (V.P.T.); tinatvi@mail.ru (V.I.T.); 2 School of Biosciences, University of Kent, Canterbury CT2 7NJ, UK; D.K.Griffin@kent.ac.uk

**Keywords:** chicken breeds, germplasm conservation, SNPs, runs of homozygosity, linkage disequilibrium

## Abstract

**Simple Summary:**

To maintain the uniqueness of conserved chicken populations of local and imported breeds is of great importance. In this study, we genotyped small populations belonging to 14 breeds and 7 crossbreds using an Illumina Chicken 60K SNP (Single Nucleotide Polymorphisms) BeadChip and looked for appropriate methods to characterize their purity/variability. It was not straightforward to identify crossbred individuals, and the best approach was based on calculating the length and number of homozygous regions, or runs of homozygosity (ROH), in the populations studied. The latter enabled most accurate identification of crossbreds and can be served as an effective tool in testing genome-wide purity of chicken breeds.

**Abstract:**

Preserving breed uniqueness and purity is vitally important in developing conservation/breeding programs for a germplasm collection of rare and endangered chicken breeds. The present study was aimed at analyzing SNP genetic variability of 21 small local and imported purebred and F_1_ crossbred populations and identifying crossbreeding events via whole-genome evaluation of runs of homozygosity (ROH). The admixture models more efficiently reflected population structure, pinpointing crossbreeding events in the presence of ancestral populations but not in their absence. Multidimensional scaling and *F*_ST_-based analyses did not discriminate properly between purebred populations and F_1_ crossbreds, especially when comparing related breeds. When applying the ROH-based approach, more and longer ROHs were revealed in purebred individuals/populations, suggesting this as an effective implement in genome-wide analysis of germplasm breed purity.

## 1. Introduction

Assessment of the genome-wide diversity plays a significant role in conserving local and imported genetic resources and maintaining an effective program for breeding commercial populations (e.g., [1,2,3]). Previously, in the framework of a poultry germplasm conservation and utilization research project, we assessed a broad spectrum of the world’s chicken germplasm populations for genetic variation at economically important loci [4,5] and multiple single nucleotide polymorphisms (SNP) loci [6,7,8,9].

A comparative genetic evaluation of livestock populations of different origin, population history, and size is an important source of information for genetic changes in their genome, including degree of homozygosity across genome-wide regions [2,3,10]. This is especially desirable when breeding small conserved groups and evaluating crossbreeding/inbreeding effects [11,12]. Lengths of runs of homozygosity (ROH) in the genome of a particular animal eventually depend on selection, gene drift, and herd size in original population [12], and are useful for assessing information about the degree of inbreeding. Long ROHs are typical for inbred individuals, as haplotypes inherited from a common ancestor do not shorten during recombination. In contrast, short ROHs can inform studies of less pronounced inbreeding intrinsic to heterogeneous animals [13,14,15,16].

Since relevant ROH studies in small closed populations kept in a germplasm collection of local, rare, and endangered chicken breeds are missing, the goal of our study was a genome-wide evaluation of SNP genetic diversity in these populations, and a search for criteria to identify occasional hybridization events in population history in order to control breed purity. Herewith we investigated ROHs and linkage disequilibrium (LD) and hypothesized that, when monitoring intra-breed genetic structure, small purebred populations would have more and longer ROHs compared to F_1_ crossbreds, which could be served as a marker to detect potential occasional hybridization events.

## 2. Materials and Methods

For this study, SNP genotypes of 673 birds were obtained for the following 21 small populations of local and imported breeds that represent a broad sample of the world’s chicken germplasm and are maintained at the RRIFAGB Collective Use Centre ‘Genetic Collection of Rare and Endangered Chicken Breeds’. These included 14 purebred populations: Amroks Cuckoo (A), Brahma Buff (BB), Brahma Light (BL), Bantam Mille Fleur (or Russian Korolyok) (BMF), White Cornish (C; two-way hybrid C1 bred *inter se*), Frizzle (F), Hamburg Silver Spangled Dwarf (HSSD), Leghorn Light Brown (or Italian Partridge) (LLB), Pushkin (P), Russian White (two populations, RW1 and RW2), Sussex Light (SL), Tsarskoye Selo (or Tsarskoselkaya) (TC), Uzbek Game (UG); and 7 groups of F_1_ crossbred progeny: Brahma Light × Sussex Light (BL × SL), Sussex Light × Amroks Cuckoo (SL × A), Tsarskoye Selo × Sussex Light (TC × SL), Uzbek Game × Amroks Cuckoo (UG × A), White Cornish × (Brahma Light × Sussex) (C × BL × SL), White Cornish × (Sussex × Amroks Cuckoo) (C × SL × A), and Tsarskoye Selo × (Sussex Light × Amroks Cuckoo) (TC × SL × A) as shown in Table 1.

In each population, both hens and cocks were genotyped using Illumina Chicken 60K SNP BeadChip (Illumina, USA) as performed with the assistance of the Geneseek/Neogen Corporation. SNPs were filtered and removed from the further analysis if they met the following criteria: minor allele frequency ≤0.05, Hardy–Weinberg equilibrium probability ≤1 × 10^−4^, and call rate ≤95%. After completing the quality control procedures, 44,230 SNP markers from 28 autosomes were available for further analysis.

LD (or *r*^2^) coefficients were calculated using the following formula in PLINK 1.9 software [17]:

(1) $$r^{2}=\frac{{\left(f_{11}f_{22}-f_{12}f_{21}\right)}^{2}}{fA_{1}fA_{2}fB_{1}fB_{2}}$$

where A and B are two loci each containing two alleles A_1_ and A_2_, and B_1_ and B_2_; *f*_11_, *f*_12_, *f*_21_ and *f*_22_ are the frequencies of haplotypes A_1_B_1_, A_1_B_2_, A_2_B_1_ and A_2_B_2_, respectively; and *f*A_1_, *f*A_2_, *f*B_1_ and *f*B_2_ are the frequencies of alleles A_1_, A_2_, B_1_ and B_2_, respectively.

Search for homozygous regions was carried out using the PLINK 1.9 program according to the following algorithm: ROHs containing at least 100 SNPs and a total length of ≥1000 Kb were taken into account, each ROH having had at least one SNP per 50 Kb. The scan window contained 50 SNPs and no more than 1 heterozygous genotype.

Values of ROH-based inbreeding coefficients (*F*_ROH_) were found using the following formula [18]:

(2) $$F_{\mathrm{ROH}}=\frac{{\sum^{\;}}_{k}\;length\left(ROH_{k}\right)}{L}$$

where *k* is the number of ROHs, *length (ROH_k_)* is an individual ROH region length, and *L* is autosomal genome size covered by SNPs.

Population variability of SNP markers in purebred and F_1_ crossbred populations was estimated in the admixture 1.3.0 program [19]. To compute the maximum likelihood estimates, the most probable number of ancestral populations (K) was selected using genotype data from 44,230 SNPs. For this purpose, a cross-validation procedure was performed. The lowest cross-validation error was found at K = 10, and charts were plotted using the function barplot() in R [20].

Multidimensional scaling (MDS) was performed in Plink 1.9 software [21] using a matrix of identity by state (IBS) distances between samples. To eliminate the effect of individual’s sex on IBS distances, SNP markers located on the sex chromosomes were excluded from the MDS analysis and, therefore, genotype data from 44,230 autosomal SNPs were employed.

Additionally, to assess between population diversity, Wright’s [22] fixation index (*F*_ST_) values were computed using eigensoft 6.1.4 software [23]. Phylogeny tree was plotted using Neighbor Joining algorithm in PHYLIP (PHYLogeny Inference Package) [24] and Interactive Tree Of Life (iTOL v4) [25].

## 3. Results and Discussion

**Figure 1:**
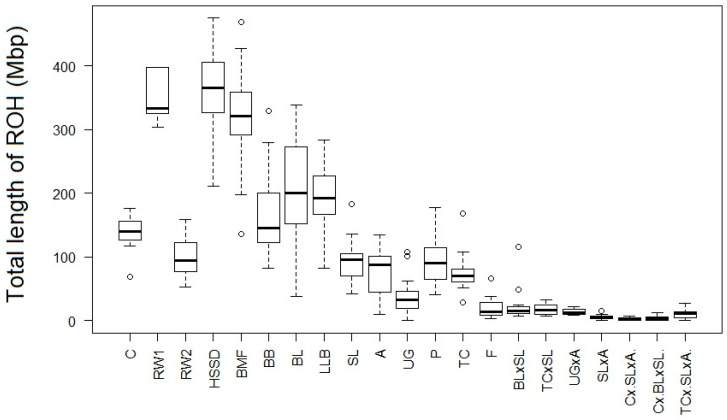
Total length of ROH (Mb) within the 21 purebred and F_1_ crossbred chicken populations studied. Abbreviations of all populations are given in Materials and Methods.

Using whole-genome SNP genotyping, we found that the examined populations of local and imported chicken breeds significantly differed from each other in genetic architecture and total length of ROHs (Figure 1). Most conserved purebred populations had greater numbers of ROHs and longer ROHs as well as higher ROH-based inbreeding coefficients (*F*_ROH_) than F_1_ inter-breed crossbreds produced by crossing two or three breeds (Table 1). For example, HSSD, an old Dutch breed that underwent a strict intra-breed type of breeding, was characterized by a greater number of ROHs (63.9 ± 1.5 vs. 32.0 ± 5.2 averaged across other 13 breed populations) and a greater length of ROH (5735.0 ± 164.2 Kb vs. an average of 4193.8 ± 182.7 Kb), with the inbreeding coefficient *F*_ROH_ being the highest one (0.324 ± 0.011 vs. an average of 0.141 ± 0.023; Table 1). All this suggests that this breed and its particular small population were affected by inbreeding both in earlier times and more recently. The presence of SNPs with higher rates of LD (0.438 ± 0.001; Table 1) also testifies to inbreeding effects in this population.

**Figure 2:**
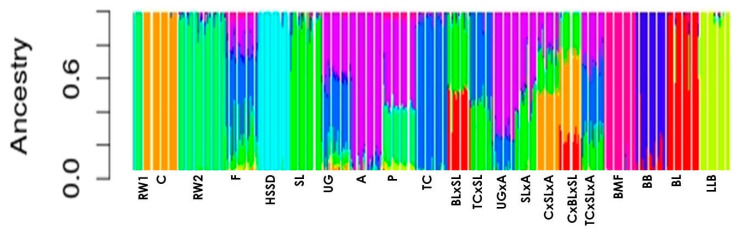
Estimation of individual ancestry from the tested SNP marker datasets as an indicator of population homogeneity/variability in the 21 purebred and F_1_ crossbred chicken populations studied as computed in the admixture program (K = 10). Abbreviations of all populations are given in Materials and Methods.

There were other small closed purebred populations with a higher content of ROHs in their genome, e.g., BB (*F*_ROH_ = 0.167 ± 0.015), BMF (*F*_ROH_ = 0.291 ± 0.015), etc. (Table 1). Among these, we found an exception with *F*_ROH_ = 0.023 ± 0.005 in the decorative F breed known in Europe since 1676 and characterized by a specific structure of feathers due to the *F* (frizzling) gene. A lower content of ROHs in this breed population can be associated with a targeted introgression of genes of the other breeds to reduce the effect of feather fragility as reflected by the admixture analysis (Figure 2).

The original Russian White population (RW1) existed from 1953–2003, had one founder and underwent intensive selection for chick tolerance to cold [7,26]. In 2005, in order to maintain genetic diversity in a small population of this breed, a single introductory crossing with White Leghorns resulted in producing the present population RW2. Accordingly, we observed a higher *F*_ROH_ in RW1 (0.307 ± 0.014) and a lower one in RW2 (0.083 ± 0.006; *p* < 0.0001; Table 1). Also, these two populations differed in LD, with a lower value being in RW2 as compared to RW1 (*p* < 0.0001; Table 1). Despite a relatively small size (~200 animals), RW2 is not expected to raise homozygosity because of the previous crossing with White Leghorns that was likely to significantly increase its genetic diversity.

In two-breed F_1_ crossbreds, we revealed a decrease in ROH metrics, while three-breed F_1_ crossbreds appeared to show a further lowering in their genomic content of ROH (Table 1).

Number of ROHs could reflect the type of breeding program. Small local and imported conserved populations bred *inter se* had a greater (>20) number of ROHs (e.g., BMF, BB, BL, RW1 and C), meaning a reduced genetic diversity similar to what was shown in small livestock populations kept by individual breeders or in local breeds [2,27,28,29,30,31]. In contrast, the genomes of two- and three-breed F_1_ crossbred progenies contained a lesser (<6) number of ROHs (Table 1; Figure 1), which was consistent with lower ROH metrics observed in other studies on crossbred animals (e.g., [3,32]).

**Figure 3:**
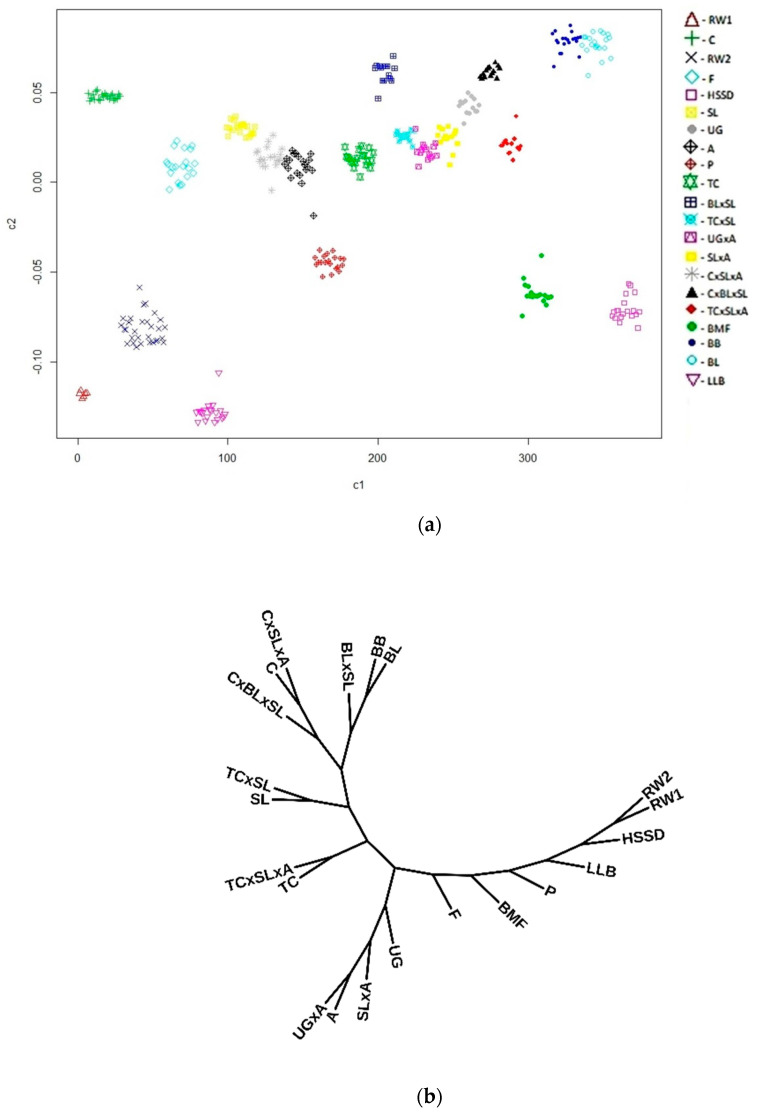
Distribution of the 21 chicken populations studied on the basis of genotype data from 44,230 SNPs and values of (**a**) identity by state (IBS) matrix (Multidimensional scaling (MDS) plot using the first two principal components, c1 and c2) and (**b**) FST matrix (Neighbor Joining tree) that did not clearly discriminate between purebred and F1 crossbred populations. Abbreviations of all populations are given in Materials and Methods. There are many chicken breeds/populations in the world that could be similar in phenotype, but different in origin. This may cause a problem of identification and discrimination between breeds/populations, which can be solved with a high accuracy by comparing them against the available global database of SNP genotypes. The differentiation of individuals and their affiliation to a certain population or populations can be assessed using the MDS method. The accuracy of this analysis is significantly affected by the genetic background of populations selected for comparison, the breeding history of populations, and their effective population size. Therefore, there may be a bias in the assessment or false conclusions. For example, the distribution of F1 crossbreds in Figure 3a does not directly reflect their origin. The tree topology based on FST analysis (Figure 3b) also does not clearly suggest the original breeds used for producing F1 crossbreds.

Characterization of genetic differentiation in conserved populations of local and imported breeds and monitoring of their purity/variability are an important component of breeding/conservation programs [1,2]. In the presence of ancestral purebred populations, use of the admixture program effectively detects F_1_ crossbred progenies (Figure 2). However, in the absence of ancestral populations, it is difficult to determine whether it is an F_1_ crossbred progeny or a purebred population (data not shown). If data on the origin are not taken into account, F_1_ crossbred progenies are difficult to identify. In contrast, when conventional MDS and *F*_ST_-based analyses (Figure 3a,b) are performed, one cannot always describe properly the population structure, differentiation, and gene flow due to crossbreeding, especially when comparing related breeds.

## 4. Conclusions

Exploration of the length and number of ROHs can be helpful in discriminating between purebred and F_1_ crossbred animals, and this approach can be used as a tool in selecting purebred individuals for conserved propagation of local and imported breeds. ROH-based characterization in individuals and in the whole population can be used in adjusting germplasm breeding/conservation programs and identifying events of inter-breed gene transfer.

## Figures and Tables

**Table 1:** Runs of homozygosity (ROH)-based metrics and linkage disequilibrium (LD) values in the 21 purebred and F_1_ crossbred chicken populations studied.

Population	Abbreviation Code	Origin	Type	Sample Size	Number of ROHs per Individual			Length of ROH, Kb			*F*_ROH_^1^	LD
					Mean	Minimum	Maximum	Mean	Minimum	Maximum		
*Pure breeds*												
Amroks Cuckoo	A	USA	dual purpose	20	18.9 ± 1.5	3	31	4057.2 ± 198.8	2308.9	5998.3	0.105 ± 0.009	0.187 ± 0.001
Brahma Buff	BB	India, USA	fancy, meat	20	36.6 ± 2.1	24	56	4508.4 ± 194.7	3116.2	6347.6	0.167 ± 0.015	0.283 ± 0.001
Brahma Light	BL	“ ^2^	“	20	37.4 ± 3.0	10	56	5259.3 ± 193.4	3772.7	6916.0	0.203 ± 0.017	0.286 ± 0.001
Bantam Mille Fleur (or Russian Korolyok)	BMF	Russia	fancy	20	61.2 ± 2.1	31	78	5259.0 ± 234.0	4043.3	8082.3	0.291 ± 0.015	0.374 ± 0.001
White Cornish (two-way hybrid C1 bred *inter se*)	C	England	meat	20	40.4 ± 1.1	33	52	3609.8 ± 80.7	3105.0	4494.0	0.055 ± 0.007	0.155 ± 0.001
Frizzle	F	Asia, Europe	fancy	20	5.9 ± 0.1	1	20	3149.0 ± 234.1	1394.6	6322.4	0.023 ± 0.005	0.171 ± 0.0003
Hamburg Silver Spangled Dwarf	HSSD	Holland	“	20	63.9 ± 1.5	42	76	5735.0 ± 164.2	4672.2	7311.6	0.324 ± 0.011	0.438 ± 0.001
Light Brown Leghorn (or Italian Partridge)	LLB	Italy	egg	19	47.5 ± 2.3	26	62	3903.8 ± 137.7	2949.8	5033.0	0.167 ± 0.011	0.288 ± 0.001
Pushkin	P	Pushkin, USSR/Russia	dual purpose	20	23.8 ± 1.3	15	36	3889.4 ± 209.7	2449.5	5569.3	0.112 ± 0.009	0.232 ± 0.001
Russian White	RW1	Pushkin, Russia	egg	6	68.3 ± 2.0	62	76	5121.9 ± 278.3	4279.8	5850.7	0.307 ± 0.014	0.518 ± 0.001
Russian White	RW2	“	“	170	25.9 ± 1.1	16	36	3749.6 ± 111.3	2431.3	4737.0	0.083 ± 0.006	0.218 ± 0.001
Sussex Light	SL	England	dual purpose	20	23.1 ± 1.5	11	35	4075.4 ± 155.8	3030.5	5773.5	0.127 ± 0.003	0.263 ± 0.001
Tsarskoe Selo (Tsarskoselskaya)	TC	Pushkin, Russia	“	20	18.6 ± 1.2	8	30	4098.5 ± 193.1	2765.8	6303.2	0.102 ± 0.009	0.201 ± 0.001
Uzbek Game	UG	Uzbekistan	game	19	9.0 ± 1.3	0	21	3838.4 ± 349.5	0	6215.3	0.095 ± 0.007	0.178 ± 0.001
*F_1_ crossbreds*												
Brahma Light × Sussex Light	BL × SL	Pushkin, Russia	meat	12	4.3 ± 0.5	1	7	3602.2 ± 378.5	2347.8	7266.8	0.051 ± 0.008	0.215 ± 0.001
Sussex Light × Amroks Cuckoo	SL × A	“	“	14	2.2 ± 0.3	0	5	2905.1 ± 291.9	0	5126.7	0.033 ± 0.008	0.188 ± 0.001
Tsarskoye Selo × Sussex Light	TC × SL	“	“	14	6.0 ± 0.7	3	10	3032.8 ± 188.2	2050.0	4181.1	0.040 ± 0.009	0.215 ± 0.001
Uzbek Game × Amroks Cuckoo	UG × A	“	“	14	4.9 ± 0.4	3	8	3023.5 ± 170.8	2062.4	4098.3	0.039 ± 0.008	0.206 ± 0.001
White Cornish × (Brahma Light × Sussex Light)	C × BL × SL	“	“	14	0.9 ± 0.3	0	3	1622.8 ± 457.5	0	4427.7	0.024 ± 0.008	0.206 ± 0.001
White Cornish × (Sussex Light × Amroks Cuckoo)	C × SL × A	“	“	14	1.4 ± 0.4	0	4	1840.6 ± 425.2	0	4641.6	0.033 ± 0.008	0.193 ± 0.001
Tsarskoye Selo × (Sussex Light × Amroks Cuckoo)	TC × SL × A	“	“	14	3.8 ± 0.6	0	8	2715.2 ± 383.2	0	5410.2	0.046 ± 0.008	0.195 ± 0.001

^1^*F*_ROH_, ROH-based inbreeding coefficient; ^2^ As above.

## References

[1] Romanov M.N., Weigend S., Dekkers J.C.M., Lamont S.J., Rothschild M.F. (1999). Genetic diversity in chicken populations based on microsatellite markers. Proceedings of the Conference from Jay Lush to Genomics: Visions for Animal Breeding and Genetics.

[2] Chen L., Wang X., Cheng D., Chen K., Fan Y., Wu G., You J., Liu S., Mao H., Ren J. (2019). Population genetic analyses of seven Chinese indigenous chicken breeds in a context of global breeds. Anim. Genet..

[3] Joaquim L.B., Chud T.C.S., Marchesi J.A.P., Savegnago R.P., Buzanskas M.E., Zanella R., Cantao M.E., Peixoto J.O., Ledur M.C., Irgang R. (2019). Genomic structure of a crossbred Landrace pig population. PLoS ONE.

[4] Lee M.O., Romanov M.N., Plemyashov K.V., Dementieva N.V., Mitrofanova O.V., Barkova O.Y., Womack J.E. (2017). Haplotype structure and copy number polymorphism of the beta-defensin 7 genes in diverse chicken breeds. Anim. Genet..

[5] Dementieva N.V., Fedorova E.S., Krutikova A.A., Mitrofanova O.V., Stanishevskaya O.I., Pleshanov N.V., Smaragdov M.G., Kudinov A.A., Terletsky V.P., Romanov M.N. (2020). Genetic variability of indels in the prolactin and dopamine receptor D2 genes and their association with the yield of allanto-amniotic fluid in Russian White laying hens. J. Agric. Sci..

[6] Romanov M.N., Dementyeva N.V., Terletsky V.P., Plemyashov K.V., Stanishevskaya O.I., Kudinov A.A., Perinek O.Y., Fedorova E.S., Larkina T.A., Pleshanov N.V. (2017). Applying SNP array technology to assess genetic diversity in Russian gene pool of chickens. Proceedings of the International Plant and Animal Genome XXV Conference.

[7] Dementeva N.V., Romanov M.N., Kudinov A.A., Mitrofanova O.V., Stanishevskaya O.I., Terletsky V.P., Fedorova E.S., Nikitkina E.V., Plemyashov K.V. (2017). Studying the structure of a gene pool population of the Russian White chicken breed by genome-wide SNP scan. Selskokhoziaĭstvennaia Biol..

[8] Dementeva N.V., Kudinov A.A., Mitrofanova O.V., Mishina A.I., Smaragdov M.G., Yakovlev A.F. (2018). Chicken resource population as the source of study genetic improvement of indigenous breeds. J. Anim. Sci..

[9] Kudinov A.A., Dementieva N.V., Mitrofanova O.V., Stanishevskaya O.I., Fedorova E.S., Larkina T.A., Mishina A.I., Plemyashov K.V., Griffin D.K., Romanov M.N. (2019). Genome-wide association studies targeting the yield of extraembryonic fluid and production traits in Russian White chickens. BMC Genom..

[10] Zhang Q., Guldbrandtsen B., Bosse M., Lund M.S., Sahana G. (2015). Runs of homozygosity and distribution of functional variants in the cattle genome. BMC Genom..

[11] Purfield D.C., Berry D.P., McParland S., Bradley D.G. (2012). Runs of homozygosity and population history in cattle. BMC Genet..

[12] Peripolli E., Munari D.P., Silva M.V.G.B., Lima A.L.F., Irgang R., Baldi F. (2016). Runs of homozygosity: Current knowledge and applications in livestock. Anim. Genet..

[13] Kirin M., McQuillan R., Franklin C.S., Campbell H., McKeigue P.M., Wilson J.F. (2010). Genomic runs of homozygosity record population history and consanguinity. PLoS ONE.

[14] Bosse M., Megens H.J., Madsen O., Paudel Y., Frantz L.A.F., Schook L.B., Crooijmans R.P., Groenen M.A. (2012). Regions of homozygosity in the porcine genome: Consequence of demography and the recombination landscape. BMC Genet..

[15] Herrero-Medrano J.M., Megens H.J., Groenen M.A.M., Ramis G., Bosse M., Perez-Enciso M., Crooijmans R.P. (2013). Conservation genomic analysis of domestic and wild pig populations from the Iberian Peninsula. BMC Genet..

[16] Ceballos F.C., Joshi P.K., Clark D.W., Ramsay M., Wilson J.F. (2018). Runs of homozygosity: Windows into population history and trait architecture. Nat. Rev. Genet..

[17] Thomas L., Ferreira M.A., Bender D., Maller J., Sklar P., de Bakker P.I., Daly M.J., Sham P.C. (2007). PLINK: A tool set for whole-genome association and population-based linkage analyses. Am. J. Hum. Genet..

[18] McQuillan R., Leutenegger A.-L., Abdel-Rahman R., Franklin C.S., Pericic M., Barac-Lauc L., Smolej-Narancic N., Janicijevic B., Polasek O., Tenesa A. (2008). Runs of homozygosity in European populations. Am. J. Hum. Genet..

[19] Alexander D.H., Novembre J., Lange K. (2009). Fast model-based estimation of ancestry in unrelated individuals. Genome Res..

[20] Hornik K. The R FAQ (2017). https://CRAN.R-project.org/doc/FAQ/R-FAQ.html.

[21] Chang C.C., Chow C.C., Tellier L.C., Vattikuti S., Purcell S.M., Lee J.J. (2015). Second-generation PLINK: Rising to the challenge of larger and richer datasets. Gigascience.

[22] Wright S. (1978). Variability within and among Natural Populations. Evolution and the Genetics of Populations.

[23] Patterson N., Price A.L., Reich D. (2006). Population structure and eigenanalysis. PLoS Genet..

[24] Felsenstein J. (1989). PHYLIP−Phylogeny Inference Package (Version 3.2). Cladistics..

[25] Letunic I., Bork P. (2019). Interactive Tree of Life (iTOL) v4: Recent updates and new developments. Nucleic Acids Res..

[26] Sokolova A.N. (1999). Genetic and Selection Methods of Creation of a Chicken Population with an Increased Resistance to Neoplasms: Author’s Abstract. Ph.D. Thesis.

[27] Mastrangelo S., Ciani E., Sardina M.T., Sottile G., Pilla F., Portolano B. (2018). Runs of homozygosity reveal genome-wide autozygosity in Italian sheep breeds. Anim. Genet..

[28] Mastrangelo S., Tolone M., Di Gerlando R., Fontanesi L., Sardina M.T., Portolano B. (2019). Genomic inbreeding estimation in small populations: Evaluation of runs of homozygosity in three local dairy cattle breeds. Animal.

[29] Bortoluzzi C., Crooijmans R.P.M.A., Bosse M., Hiemstra S.J., Groenen M.A.M., Megens H.-J. (2018). The effects of recent changes in breeding preferences on maintaining traditional Dutch chicken genomic diversity. Heredity.

[30] Cardoso T.F., Amills M., Bertolini F., Rothschild M., Marras G., Boink G., Jordana J., Capote J., Carolan S., Hallsson J.H. (2018). Patterns of homozygosity in insular and continental goat breeds. Genet. Sel. Evol..

[31] Grilz-Seger G., Druml T., Neuditschko M., Dobretsberger M., Horna M., Brem G. (2019). High-resolution population structure and runs of homozygosity reveal the genetic architecture of complex traits in the Lipizzan horse. BMC Genom..

[32] Howard J.T., Tiezzi F., Huang Y., Gray K.A., Maltecca C. (2016). Characterization and management of long runs of homozygosity in parental nucleus lines and their associated crossbred progeny. Genet. Sel. Evol..

